# Why are antiretroviral treatment patients lost to follow-up? A qualitative study from South Africa

**DOI:** 10.1111/j.1365-3156.2010.02514.x

**Published:** 2010-06

**Authors:** Candace M Miller, Mpefe Ketlhapile, Heather Rybasack-Smith, Sydney Rosen

**Affiliations:** 1 Center for Global Health and Development, Boston UniversityBoston, MA, USA; 2 Health Economics and Epidemiology Research Office, Wits Health Consortium, Themba Lethu ClinicJohannesburg, South Africa

**Keywords:** antiretroviral therapy, South Africa, loss to follow-up, qualitative research

## Abstract

**Objectives:**

To better understand the reasons why patients default from antiretroviral treatment (ART) programmes to help design interventions that improve treatment retention and ultimately, patient outcomes.

**Methods:**

Prospective cohort study at two treatment sites in South Africa followed by qualitative interviews with patients that had defaulted.

**Results:**

Respondents overwhelmingly reported that ART improved their health status and quality of life. Nevertheless, despite improved health from taking ART and worse health when treatment is stopped, serious barriers to treatment remained: transport costs, time needed for treatment, and logistical challenges were barriers to treatment, whereas stigma around HIV/AIDS, and side effects associated with ART were less influential.

**Conclusion:**

With a better understanding of the reasons for defaulting, interventions can be designed that improve treatment retention and ultimately, patient outcomes. This study argues for realistic interventions and policy changes designed to reduce the financial and time burden of ART and to reduce logistical barriers, such as simplifying the referral and transfer process, employing patient advocates, and adopting extended and weekend clinic hours.

## Introduction

As large-scale programmes to provide antiretroviral treatment (ART) for HIV/AIDS have expanded and matured in sub-Saharan Africa, attention has shifted from a single-minded focus on treatment access and initiation to the broader set of long-term challenges in sustaining a vast and complicated public health endeavour. One of these concerns is retention of patients in care. ART is a lifelong commitment that requires patients to adhere diligently to daily medication dosing schedules and make frequent clinic visits for care. Consistent with experiences in treating chronic diseases globally, a systematic review of patients who initiated ART across sub-Saharan Africa found that approximately 25% were no longer in care 1 year after initiation, a figure rising to 40% after 2 years (Rosen *et al.* 2007). Among this group of patients, a minority had died, while the majority was classified as ‘lost to follow up’.

Patients who discontinue treatment are at high risk of illness and death because of AIDS-related conditions. Consequently, many studies have attempted to quantify and ascertain the status of patients reported as lost to follow-up, including several in South Africa (MacPherson *et al.* 2009, Dahab *et al.* 2008; Maskew *et al.* 2007). At a large public hospital, 31% of patients who defaulted soon after ART initiation had died, 25% had transferred and 44% had discontinued treatment voluntarily or could not be found (Dalal *et al.* 2008). Another public sector clinic in South Africa obtained similar results after tracing patients who were at least 1 month late for their last clinic visit 6 months after starting ART: 41% had died, 7% had transferred, and 52% had voluntarily discontinued treatment or could not be found (Dahab *et al.* 2008). Studies from other countries report similar outcomes after actively tracing those initially reported as lost to follow-up (Billy *et al.* 2007, Bisson *et al.* 2008, Tweya *et al.* 2009, Geng *et al.* 2008, Krebs *et al.* 2008, Ochieng *et al.* 2007, Yu *et al.* 2007).

A number of reasons for defaulting from treatment programmes have been identified or hypothesized. These include cost, transport and waiting time, stigma, family pressures, religious beliefs and illness (Murray *et al.* 2009, Aidi *et al.* 2009, Roura *et al.* 2009). Numerous studies in South Africa and elsewhere have concluded that cost is a major cause of attrition in clinics, particularly those that charge fees for services (Nachega *et al.* 2004; Maskew *et al.* 2007; Dahab *et al.* 2008). Relocation is also a common reason for dropout in South Africa, where the population is highly mobile (Dahab *et al.* 2008).

To gain more insight into the reasons why patients default from treatment programmes, and to suggest actionable interventions to improve retention, we conducted a prospective cohort study at two treatment sites in South Africa, where more than half a million people were receiving ART by the end of 2008 (UNAIDS 2008). We conducted qualitative follow-up interviews with patients who had defaulted from treatment to better understand their reasons for defaulting. We conducted qualitative follow-up interviews with patients who had defaulted from treatment to better understand their reasons for defaulting. Thus, patients may themselves help to identify interventions that will foster long-term retention in care.

## Methods

The subjects from this study were enrolled in a prospective cohort of adult patients receiving ART at two treatment centres in South Africa: an NGO clinic serving a rural population in Limpopo Province and a large public hospital in Gauteng Province. Patients were eligible for enrolment if they (i) had initiated ART at least 6 months, but no more than 3 years, before study recruitment, (ii) were still on ART and (iii) were not more than 1 month late for their most recent medication pickup or consultation.

After providing written consent, a random sample of patients (*n* = 528) was enrolled in the study. Study personnel administered a baseline questionnaire during a routine clinic visit, collecting detailed contact information for the patient. Twelve months after the baseline, clinic medical records were reviewed to determine each subject's status. Status categories included (i) in care, (ii) defaulted, (iii) died and (iv) transferred. Default was defined as being more than 1 month late for the next scheduled consultation or medication pick up. Defaulting patients were contacted and interviewed either face-to-face or by telephone. Follow-up interviews were qualitative and addressed the subject's perceptions of the treatment and clinic, current health status, and reasons for stopping treatment, and also included an offer of assistance in resuming treatment if the subject desired. For this analysis, we focused solely on patients who had already defaulted because our aim was to understand the immediate obstacles facing such patients, and thus to help identify practical interventions that can improve patient retention.

Data were analysed using NVIVO 8 software. Interviews were read several times to identify major themes and ideas. We also examined the consistency of ideas and experiences within individual transcripts. Then a detailed list of categories was created to reflect the major themes that emerged from the interviews and the text from each interview transcript was coded into the various categories. Next, we examined the categories, noting the similarities and differences within each category and quantifying the frequency with which ideas occurred. We present qualitative data using patients' voices to describe their current health status and reasons for defaulting or transferring. Typical quotes are presented to illustrate the various phenomena.

The study protocol was approved by the Boston University Institutional Review Board and the Human Research Ethics Committee (Medical) of the University of the Witwatersrand.

## Results

A total of 528 subjects were enrolled in the cohort, including 291 from the urban public hospital and 237 from the rural clinic. At both sites, these patients were almost equally divided among those who had been on ART 6–12, 13–24 and 25–36 months at the time of enrolment. Twelve months after enrolment, seven subjects had died, 25 subjects had been referred from the urban hospital to a nearby primary care clinic, 16 had transferred to another treatment site and 17 were more than 1 month late for their last scheduled medication pickup or consultation and were considered to have defaulted. Patients were eligible for the qualitative study if they had defaulted on treatment for more than 1 month. At the time of the interview, defaulters were between 3 and 11 months beyond their previously scheduled appointment (median = 7 months). Transfer patients were interviewed 2–13 months after their final appointment at their initial clinic (median = 7 months). Fourteen of the defaulting patients and all 16 transfers were interviewed regarding reasons for default. Two of the defaulting subjects were not located and one refused to be interviewed. Twelve defaulters reported having stopped treatment at the time of the interview, and two reported that they had re-started treatment at the original clinic, after an interval of missed appointments.

In this analysis, we included both patients who transferred to new clinics on their own accord and patients who stopped treatment. While transfer patients may successfully remain in care at another site, their experience still provides insight into the struggles and challenges that antiretroviral (ARV) ARV patients face. We chose not to interview patients who were down-referred from the urban hospital, as good adherence and an undetectable viral load are criteria for down-referral, and these patients thus have better-than-average clinic attendance records.

In the larger random sample (*n* = 528), 75% of patients were women. Among the qualitative sample of defaulters and transfer patients, 65% were women (Table 1). In this small qualitative sample, we found that compared to transfer patients, defaulters were younger, more likely to be woman and have higher levels of schooling, and they were less likely to receive government grants or be unemployed. The default rate in the study population was similar between men (4%) and women (3%), and the transfer rate was also similar (5% for men, 4% for women).

**Table 1:** Demographic background of study respondents

	Default patients (*n* = 14)	Transfer patients (*n* = 16)	All patients (*n* = 28)
Age (mean years)	31	34	35
Gender (% male)	25	42	35
Level of schooling (% reaching matriculation or beyond)	72	25	46
Receiving government grants (%)	8	44	29
Unemployed (%)	25	44	36

### Perceptions of treatment

Patients universally reported that they had experienced important, even extraordinary health benefits from taking ART and recognized that stopping ART would lead to illness. Weight gain and improved mobility were commonly noted:

The ARVs made me gain back my weight…even if I have not gained it very much, they enabled me to wake up again… (Female 31, defaulter 9 months)

I don't know where to start when it comes to ARVs because I feel I'm a person (again)…, People who saw me when I was ill cannot believe what they see (now). (Female 31, transfer patient)

The ARVs worked for me because they brought me back to life, I feel different than before. At that stage I was very, very, very sick…, so they gave me back my life. (Female 36, defaulter 4 months)

Subjects also showed an understanding of the relationships between ART and health, ART and CD4 counts, and CD4 counts and illness.

ARVs are fine if taken properly… I am sick because I stopped. (Male 27, defaulter 10 months)

The difference is that when I was taking ARVs, I was not sick, but now I am starting to feel a bit sick, my bones and my arm are not well. (Male 40, defaulter 4 months)

Before I took them I was weak, I could not do anything, take a walk like other people…now my immune system is high…it is 800 and something… from 123 in 2005. (Male 48, transfer patient)

Depending on their CD4 I will tell them to go and start medication, and if they are sick, but if it is not too low it is fine not to start. (Male 34, defaulter 11 months)

In addition to improved health because of ARVs and understanding the relationship between treatment and health, most respondents reported that they would refer family and friends for ART if needed. The respondents that reported that they would advise others to take ARVs if needed also demonstrated a high level of understanding of the importance of remaining on treatment and taking pills on time.

I will say that because I was sick, I was lying down because of my sickness… and I woke up again, I will tell them to drink ARVs in the right way and on time. (Female 35, transfer patient)

Because I saw they work, they have power, as I was told, and to use a condom and drink them accordingly. (Male 29, transfer patient)

### Current physical and mental health status

Among defaulting patients, seven of 14 reported experiencing negative physical health effects since stopping treatment, although some reports were minor, such as a worsened cough.

I left work actually early today, I was not feeling well, I felt dizzy…, yes, it is also how I used to feel when I started realizing I am HIV positive… I also had some diarrhoea a bit since yesterday …(Male, 27, defaulter 10 months)

I'm fine but not so fine, my feet are painful and I have a little coughing, I cannot go up the stairs, and it is because I am not taking ARVs. When I was taking ARVs I was fine. (Female, 33, defaulter 8 months)

I have not been well for the past 2 weeks, even now, it is my temperature and I have headaches …(Female, 32, defaulter 2 months)

I don't have anything that is a problem with my health, I am still fine. (Female, 36, defaulter 4 months)

A number commented about feeling stressed or worried, because of pressures at home or at work or concern about having stopped treatment.

My stress is that I cannot come to collect my treatment…there is no pain in the my body, the problem is I work in a mine and I cannot come to the clinic because it is far (Male 40, defaulter 6 months)

Others reported, however, that they felt well both physically and mentally.

All in all I am happy, except for some small things that can cause stress…like employment, I have a lot of work to do and I cannot cope… but I am generally happy. (Female 24, defaulter 4 months)

### Reasons for defaulting

All interviewees reported disclosure of their status to others. In fact, all respondents, except for one transfer patient, reported that they had someone in their household who knew about their treatment. Respondents rarely reported stopping medication because of family pressure or stigma. Although one subject indicated that fear of stigma in the workplace contributed to his inability to request time off to collect medication, another respondent stated that pressure and fear of disclosure did not affect her decision:

One of the reasons I stopped treatment might be because of [transportation] costs, but not side effects or fear of disclosure, because there are people in my street who know my status, and my friends. No, no one has pressurized me to stop my pills. (Female 31, defaulter 9 months)

Although the treatment sites did not charge for care, several subjects had difficulty with transportation costs. Of the 20 patients who transferred to new treatment sites, 16 had lower transport costs to get to the new clinic. Reduced transport costs and time were the main reported reasons for transferring. Transfer patients were more likely to be unemployed and be receiving government grants than defaulters, suggesting that transport costs were indeed an important consideration. Transfer patients spent R22 (range R10–R200) on transportation at baseline, a cost that fell to R12 per visit (range R0–R46) at the new clinic. The median spent on transportation by patients who defaulted was R13 (range R0–R72).

Of the 14 defaulted patients, seven reported that employment caused difficulties with obtaining medication. Five reported that they could not take time off during the regular clinic operating hours. Four reported a missed appointment or medication pickup because of travel for work or family-related events.

I was not able to go to the hospital because I started at a new company and I could not take off…clinics must be open over the weekend for people who work … most people who do not turn up for their appointments are working. (Male 34, defaulter 11 months)

I am 4 months without taking treatment, the main reason being that I work Monday till Saturday, I don't have any other reason. Before I worked for a different security company, and I could take off for 3 or 4 days, with this company there is no offs. (Female 31, defaulter 4 months)

Several patients cited missing paperwork, including clinic cards, transfer papers, and proof of travel, as a reason for missed appointments.

I lost my card and I was afraid to come to the clinic … isn't it that at the reception when you have no card they tell you to go back …(Female 32, defaulter 2 months)

Several subjects reported abandoning treatment because of long wait times, and there were reports of other health systems concerns. Among the issues reported were long queues, difficulty in booking appointments, difficulty tracking down paperwork or staff, too many patients at clinics, not enough time spent with providers, and clinics running low on medication.

You see, Sisi, when you go to the hospital you have to book … next you go to the clerks, but every time there is a misunderstanding, they do not book you on the appointment you agreed on. Even when you try to make an appointment on the telephone, you never know if they took your file out to book you on the date you requested or not. And when you go back you wait until 4 o'clock, they start telling you that you did not book. (Female 36, defaulter 4 months)

The thing that is making me not to go to the clinic is because my file is held by the social worker, I have been there twice, I do not have money to be going up and down and I walk there and back. If I want money they (social workers) do not give me. (Female 33, defaulter 8 months)

About the pills, sometimes they say there are no pills and send me to the hospital but I am persevering, I go there. (Female 37, defaulted on clinic visits 8 months, but reported obtaining pills from another clinic)

They do not have the time for people, they only give you what you need and then you go … (Female 36, transfer patient)

Only two defaulted and five transferred patients reported side effects. Of these, three complained of extremity pain, one of eye discomfort and two of body shape changes. Two women reported that side effects (body shape changes) might be a contributing factor to default, although this was not reported as a major barrier to treatment.

But they have advantages and disadvantages … there are things that are hidden, like they deform you, they changed my structure. (Female 36, defaulter 4 months)

[Regarding reasons for stopping ART] It can be because of side effects with my breasts, they grew very big (Female 32, defaulter 2 months)

The use of traditional medicine was not a major theme in respondent reports, but it was mentioned by two subjects as contributing to their decision to stop treatment. One subject had stopped taking pills while in training to become a sangoma (traditional healer).

I am not taking ARVs, I am just buying and drinking my immune boosters … (Female 38, transfer patient)

I have been using Imbiza (traditional medicine) since I stopped ARVs …, it was my mother who said we must try the traditional healer because they said his Imbiza helps …(Male 27, defaulter 10 months)

Finally, the belief that a positive attitude could directly affect HIV progression and even effectiveness of ART was prevalent in the interviews.

I see them (ARVs) as right, but you know…, they go with the belief of a person and with the person's body … if a person believes that they will help they will, but if a person is not trusting them, has a negative belief about them, then they will not help them. (Female 24, defaulter 4 months)

## Discussion

We interviewed patients who had defaulted or transferred at least 6 months after initiating ART, allowing us to focus on longer-term reasons for discontinuing or changing treatment among those who were at least initially successful in their clinic attendance. Respondents overwhelmingly reported that ART improved their health status and quality of life. While most respondents associated declines in health to lack of ART, some treatment defaulters reported no ill effects from stopping ART, which may be because of the short time between defaulting and the interviews.

Nevertheless, despite improved health from taking ART and worse health when treatment is stopped, there were still serious barriers to treatment. We found that transport costs, time needed for treatment, and logistical challenges were barriers to treatment, but that stigma and side effects associated with ART were less influential.

In contrast to other studies in the literature, the cost of medication was not an important barrier among respondents because patients obtained ART and healthcare at clinics free of charge. However, transfer patients reported that transportation costs influenced treatment retention. The majority of transfer patients lowered transportation costs by changing clinic. Possible interventions to overcome financial barriers include providing stipends or vouchers to patients to cover transport costs. Additionally, a treatment plan designed to accommodate less frequent appointments and drug pick up, such as every 3–6 months, could reduce patient costs and reduce the time burden that ART places upon patients.

Both transfer patients and defaulters described logistical barriers to treatment. Many patients that had transferred to other facilities for care reported that the referral process was confusing. Without paperwork, patients encountered difficulties in transferring to new clinics, and needed to re-start treatment, placing undue burdens on both patients and clinic staff. Given that patients may need to transfer, simplifying the referral and transfer process would enable the continuation of care for patients, and minimize repetitive processes for overtaxed staff.

All patients reported challenges related to clinic operating hours, paperwork and procedures. Respondents also reported difficulty securing and changing appointments, inaccessible staff and long queues. Patients could not always determine staff roles or the required procedures. These uncertainties were insurmountable for some patients without help from the interviewer, who helped several patients return to treatment. A permanent staff member who functioned like our interviewer might help to bridge the gap between patients and staff. Extended and weekend clinic hours would enable half of the interviewees to remain compliant with ART. Even with limited resources, Saturday and extended weekday hours may be possible. Patients who travel would benefit if allowed to obtain larger supplies of medication, or to temporarily obtain mediation from another clinic. Convenient ways to obtain medication and consistent, streamlined processes would assist patients in remaining in care.

Stigma did not emerge as an important reason for defaulting from treatment. Similarly, traditional medication did not appear to interfere with ART for most patients although several patients reported taking traditional medication both in addition to and as a substitute for ART. Many patients reported feelings of stress, but few associated their stress with a reduced capacity to adhere to their medication regimen. Only one patient reported that negative side effects led her to discontinue treatment. Given the inclusion of stavudine, a drug normally associated with a high burden of toxicities, in first-line ART in South Africa, this is an unexpected finding.

We were able to locate and interview more than 90% (30/33) of patients who defaulted or transferred 1 year after study enrolment. The use of an interviewer acquainted with local customs and culture was beneficial in both the quality of data gathered and the interpretation of local euphemisms and expressions. The interviewer was not able to validate information provided by some transfer patients; however, and it is possible that some patients who reported transferring had actually stopped treatment. As the interviews were designed to investigate barriers to treatment, however, patients who were unwilling to admit default would probably still report their difficulties and struggles with adherence. Inaccurate reports of transfer might reflect deference to the interviewer or shame and embarrassment.

With more insight into the reasons for defaulting and transferring, interventions can be designed to improve ART retention and ultimately, patient outcomes. This study argues for interventions and policy changes designed to reduce the financial and time burden of ART and to reduce logistical barriers, such as simplifying the referral and transfer process, employing patient advocates, and adopting extended and weekend clinic hours.
